# Highly Selective Room-Temperature Blue LED-Enhanced NO_2_ Gas Sensors Based on ZnO-MoS_2_-TiO_2_ Heterostructures

**DOI:** 10.3390/s25061781

**Published:** 2025-03-13

**Authors:** Soraya Y. Flores, Elluz Pacheco, Carlos Malca, Xiaoyan Peng, Yihua Chen, Badi Zhou, Dalice M. Pinero, Liz M. Diaz-Vazquez, Andrew F. Zhou, Peter X. Feng

**Affiliations:** 1 Department of Physics, University of Puerto Rico, San Juan, PR 00936, USA; soraya.flores@upr.edu (S.Y.F.); elluz.pacheco@upr.edu (E.P.); 2 Department of Chemistry, University of Puerto Rico, San Juan, PR 00936, USA; carlos.malca@upr.edu (C.M.); dalice.pinero@upr.edu (D.M.P.); liz.diaz2@upr.edu (L.M.D.-V.); 3 Chongqing Key Laboratory of Brain-Inspired Computing and Intelligent Control, College of Artificial Intelligence, Southwest University, Chongqing 400715, China; pengxy2025@swu.edu.cn; 4 Department of Education, Shanghai Dianji University, Shanghai 201308, China; chenyh@sdju.edu.cn; 5 Department of Chemistry, Biochemistry, and Physics, Indiana University of Pennsylvania, Indiana, PA 15705, USA; badi.zhou@pm.me

**Keywords:** gas sensing, room-temperature sensors, NO_2_ detection, selectivity, light-enhanced sensitivity, two-dimensional nanomaterial, transition metal dichalcogenide (TMD), metal oxide semiconductor (MOS), ternary heterostructure, ZnO-MoS_2_-TiO_2_

## Abstract

This study presents the fabrication and characterization of highly selective, room-temperature gas sensors based on ternary zinc oxide–molybdenum disulfide–titanium dioxide (ZnO-MoS_2_-TiO_2_) nanoheterostructures. Integrating two-dimensional (2D) MoS_2_ with oxide nano materials synergistically combines their unique properties, significantly enhancing gas sensing performance. Comprehensive structural and chemical analyses, including scanning electron microscopy (SEM), energy-dispersive X-ray spectroscopy (EDX), Raman spectroscopy, and Fourier transform infrared spectroscopy (FTIR), confirmed the successful synthesis and composition of the ternary nanoheterostructures. The sensors demonstrated excellent selectivity in detecting low concentrations of nitrogen dioxide (NO_2_) among target gases such as ammonia (NH_3_), methane (CH_4_), and carbon dioxide (CO_2_) at room temperature, achieving up to 58% sensitivity at 4 ppm and 6% at 0.1 ppm for NO_2_. The prototypes demonstrated outstanding selectivity and a short response time of approximately 0.51 min. The impact of light-assisted enhancement was examined under 1 mW/cm^2^ weak ultraviolet (UV), blue, yellow, and red light-emitting diode (LED) illuminations, with the blue LED proving to deliver the highest sensor responsiveness. These results position ternary ZnO-MoS_2_-TiO_2_ nanoheterostructures as highly sensitive and selective room-temperature NO_2_ gas sensors that are suitable for applications in environmental monitoring, public health, and industrial processes.

## 1. Introduction

Over recent decades, developing high-performance gas sensors has been a subject of intense study, driven by global ecological challenges. Given the importance of economic feasibility and durability in often harsh environmental conditions, research on gas sensors has focused on achieving long-term stability, miniature size, high sensitivity, and fast response times. A number of materials have been explored, with hybrid structures receiving particular attention [1,2,3,4,5]. Atomically thin two-dimensional (2D) transition metal dichalcogenides (TMDs) have attracted tremendous attention due to their unique multifunctional properties and immense potential in the fields of sensing devices at elevated temperatures (above 100 °C) [6,7,8,9].

Two-dimensional nanostructures provide advantages, including nanometer-scale thickness and a high surface area with numerous active sites that accelerate the quick adsorption and reaction of target gases. Their heterogeneous (porous) surface enhances gas interactions and assists in the penetration of gases into the material, thereby boosting gas sensing performance. Theoretical research has suggested that 2D hetero-nanostructures could improve performance as compared to single-material-based devices, indicating a synergistic effect between two or more components [10,11]. For example, Ramirez et al. theoretically investigated 2D MoS_2_ layers modified with boron nitride (BN), graphene (G), and silicone [12] based on density functional theory (DFT), as implemented in the Vienna ab initio Simulation (VASP) [13,14] and Quantum Espresso (QE) packages [15]. Liu et al. reviewed that the surface chemistry and interface coupling determined the characterization and functionalities of 2D materials and heterostructures [16].

Nitrogen dioxide (NO_2_) is produced from the burning of fossil fuels. Exposure to it can cause respiratory problems, reduce respiratory defense mechanisms, and cause structural damage to the lungs. The importance of room-temperature NO_2_ gas sensors lies in their potential to provide efficient, low-power, and versatile detection of NO_2_ without the need for elevated operating temperatures. Room-temperature NO_2_ gas sensors represent a significant advancement in gas sensing technology, offering numerous benefits over traditional high-temperature sensors in environmental monitoring and public health efforts. Their importance is highlighted by the potential to enhance energy efficiency, improve safety and stability, enable rapid and sensitive detection, facilitate integration into modern electronic devices with advanced technologies, mitigate material degradation, and reduce sensor longevity caused by elevated temperatures. For example, Kočí et al. reported a new gas sensor with a sensitivity of approximately 0.1572%·ppm^−1^ for NO_2_ and 0.1884% ppm for NH_3_, based on measurements conducted at concentrations ranging from 20 ppm to 100 ppm [8]. Compared to single-component nanomaterial-based NO_2_ gas sensors, the binary nanomaterial-based NO_2_ gas sensors offer improved sensitivity, responsivity, and response times. Chao Liu et al. developed a highly sensitive and recoverable room-temperature NO_2_ gas sensor using 2D/0D MoS_2_/ZnS heterostructures with synergistic effects [17]. Zhihui Li et al. developed a room-temperature NO_2_ gas sensor based on Pt-modified MoSe_2_ nanoflowers [18].

Significant progress has been made in binary TMD-based gas sensors [19,20], with recent studies highlighting advancements in room-temperature NO_2_ detection. In 2022, Zhao et al. used edge-enriched Mo_2_TiC_2_T_x_/MoS_2_ heterostructures to develop selective NO_2_ monitoring. Short response/recovery times around 40 s/145 s at 50 ppm and 70 s/160 s at 10 ppm were obtained. The fabricated sensing device appears to have very high responsivity and stability [21]. In 2024, Zhang et al. used the synergistic effect of charge transfer and interlayer swelling in V_2_CT_x_/SnS_2_ for ultrafast and highly sensitive NO_2_ detection at room temperature. Shorter response times of less than 10 s and excellent selectivity with a response ratio > 5 have been achieved [22]. Additionally, Yin and Liu’s work on green, efficient, and controllable preparation of In_2_O_3_ uniformly modified MoS_2_ nanoflowers for either NO_2_ or methanol detection [23,24].

Based on these achievements, we have previously synthesized and studied various 2D sheets [25,26,27,28] integrated with other 2D materials or nanostructures to form binary nanocomposites, which outperform single-component nanomaterial-based counterparts. While significant work has been conducted in this area, the reported prototypes based on binary hybrid heterostructures still face challenges detecting extremely low concentrations at room temperature.

The present work extends our investigations to ternary ZnO-MoS_2_-TiO_2_ heterostructures for high-performance gas sensors by exploring p-n junctions to enhance active layers and improve sensor sensitivity and selectivity. We believe synergistic effects can be obtained by using the unique properties of each material. The integrated ternary heterostructure maximizes active sites and electron mobility and exhibits a large surface-to-volume ratio, providing an improved surface reaction for enhanced gas adsorption, a faster response/recovery behavior due to the unique morphology, and a precisely engineered bandgap for an efficient charge transfer and superior selectivity.

The study aims to build upon previous efforts by integrating 2D MoS_2_ with oxide nanomaterials to develop ternary heterostructures [29]; since each material possesses unique properties, they can collectively contribute to and enhance various functional sensing capabilities. Distinguishable differences in gas sensing performance were studied. Various low concentrations of target gases including NO_2_, NH_3_, CH_4_, and CO_2_ have been tested, with all experiments carried out at room temperature. The fabricated hybrid ZnO-MoS_2_-TiO_2_-based prototype demonstrated exceptional performance including a 58% sensitivity at 4 ppm and 6% at 0.1 ppm of NO_2_, respectively, along with excellent selective features and a short response time. DFT calculations have been employed to investigate the gas adsorption behavior of the sensor device. These studies provide insights into the preferred adsorption sites, the band structure change, and the resulting DOS property of the ZnO monolayer upon gas adsorption.

## 2. Materials and Methods

### 2.1. Preparation of ZnO-MoS_2_-TiO_2_ Heterostructures

A straightforward preparation method was adopted for the synthesis of ZnO-MoS_2_-TiO_2_ ternary heterostructures. First, a 20 nm thick layer of TiO_2_ nanoparticles (NPs) was deposited onto a Si wafer with a 300 nm thick SiO_2_ insulating layer using 60 MHz magnetron sputtering at a constant power of 200 W for 30 s. During this process, a high-purity TiO_2_ target (99.99%) was utilized along with an Ar/O_2_ gas mixture at a ratio of 30:1. The chamber maintained a base pressure of 1.5 × 10^−7^ Torr, while the working pressure during deposition was set at 8.0 × 10^−3^ Torr.

Next, the sample was transferred into the chemical vapor deposition (CVD) chamber to deposit a 500 nm thick MoS_2_ layer at 800 °C for 30 s. Detailed descriptions of TiO_2_-MoS_2_ binary synthesis and characterizations can be found in our previous works [25,26,27,28]. The formed MoS_2_ layer consisted of a large amount of MoS_2_ clusters in arbitrary orientations. The continuous MoS_2_ sheet has an average thickness of about 5 nm and a size of approximately 4 µm in diameter.

Lastly, a nanostructured ZnO layer was applied onto the surface of MoS_2_-TiO_2_ using a cost-effective spin-coating method, utilizing a commercial ZnO nanoparticle dispersion kit in a solvent with a 1 mg/mL concentration, sourced from Fisher Scientific (Hampton, NH, USA). To ensure a uniform distribution and to break up agglomerates in solvents, the ZnO solution was ultrasonicated at 200 W with a 60 Hz amplitude and 10 s intermittent pulses for two hours prior to deposition. Several coatings were applied to enhance the density of ZnO nanowires on the MoS_2_ surface, with each spin-coating cycle lasting 20 s at 2500 rpm. The ZnO layer produced by each spin-coating cycle was approximately 450 nm thick. However, a multi-spin-coating process does not result in a significantly thicker film, as the high-speed spinning progressively thins each deposited layer by removing the excess solvent. To achieve uniform layering, annealing is necessary between spin-coating cycles to dry the film. Typically, five spin-coating cycles produce a film with a thickness of approximately 2200 ± 200 nm. After annealing at 800 °C for 2 h, the films were analyzed using scanning electron microscopy (SEM), Raman scattering, and energy-dispersive X-ray spectroscopy (EDX).

### 2.2. Basic Material Characterizations

A comprehensive characterization of the ternary sample was conducted to assess its suitability for advanced sensing applications. The surface morphology of the ternary sample was analyzed using a scanning electron microscope (SEM, JEOL-6480LV JSM, JEOL Ltd., Tokyo, Japan) under a vacuum of approximately 10^−5^ torr. The SEM operated with an accelerating voltage in the range of 10–20 kV, which provided high-resolution images of the sample’s microstructural characteristics. The elemental composition of the active layers was quantitatively analyzed using energy-dispersive X-ray spectroscopy (EDX), providing accurate insights into the material’s chemical distribution.

Additionally, Raman spectroscopy (JY Horiba T64000, HORIBA France S.A.S., Palaiseau, France) and Fourier transform infrared spectroscopy (FTIR, ATR Spectrum Two, Perkin Elmer, Inc., Waltham, MA, USA) were employed to examine the structural and vibrational properties. Raman spectroscopy offered in-depth insights into molecular vibrations and crystallinity, whereas FTIR identified the material’s chemical bonds and functional groups. These complementary techniques enabled a robust and thorough assessment of the material’s physical, chemical, and structural properties. This thorough characterization is essential for enhancing the design and performance of nanostructured materials in sensing applications.

### 2.3. Gas Sensing Measurements

The gas sensing measurements were conducted using both a homemade station [30] and an advanced 4-Probe Station (LTMP-4) controlled with LabVIEW software. Electrical signals were collected using a Keithley multimeter. Additional components included a thermocouple, heaters, power supplies, electrical interfaces, pollution gas tanks, diagnostic tools, Arduino boards, as well as Agilent and Hewlett multimeters. The target gas concentration was precisely regulated using two MKS GE50A (MKS Instruments, Inc., Andover, MA, USA) mass flow controllers.

The setup featured a sensor test chamber (LTMP from MMR Technologies, Inc., Mountain View, CA, USA) equipped with adjustable tungsten tips to establish electrical connections with the samples. An HP 6212C power source provided a constant voltage and measured changes in electrical current upon gas exposure. The LabVIEW 8.5 software automated instrument control and data acquisition, recording the gas response as current (I) over time (t). Gas flow rates were regulated by MKS GE50A mass flow controllers and monitored by an MKS 946 vacuum system controller (MKS Instruments, Inc., Andover, MA, USA). Experiments were conducted under an inert nitrogen atmosphere (UHP 99.999%) and toxic gas NO_2_ (100 ppm in N_2_). Additional tests were performed using NH_3_, CH_4_, H_2_, and CO to evaluate the sensor’s response to various gases.

## 3. Results and Discussion

### 3.1. Morphologies of Surfaces of Binary and Ternary Samples

**Figure 1:**
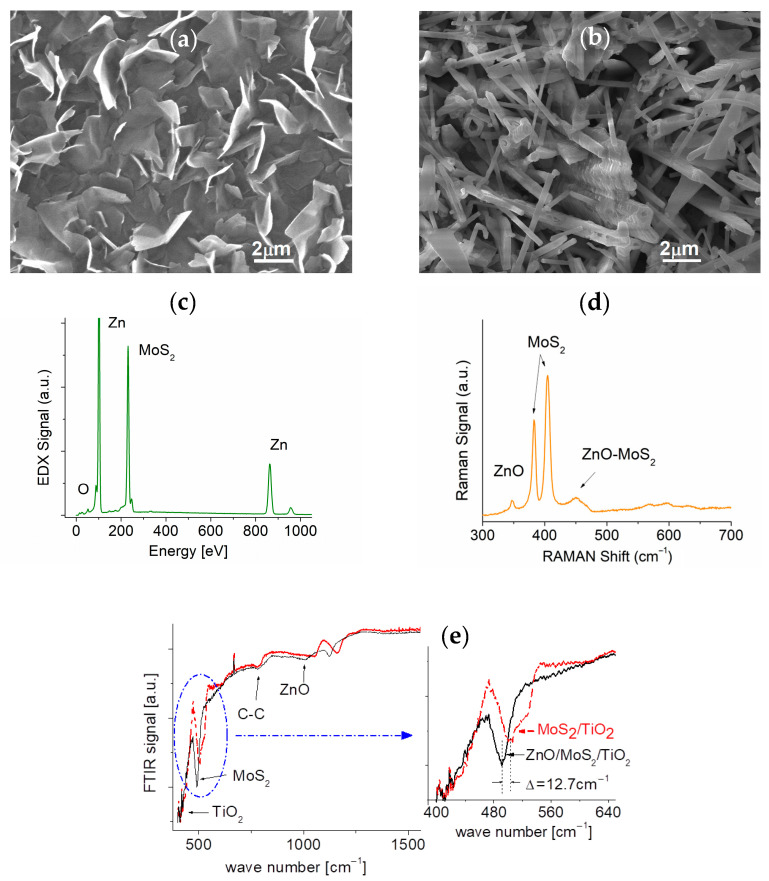
SEM images of the (**a**) MoS_2_ layer deposited on the TiO_2_-coated Si substrate and the (**b**) ZnO layer on top of the MoS_2_-TiO_2_ nanocomposite. (**c**) EDX, (**d**) Raman, and (**e**) FTIR spectrum of the ZnO-MoS_2_-TiO_2_ sample.

Figure 1a presents a representative SEM image of the surface of a binary MoS_2_-TiO_2_ sample prior to the deposition of ZnO nanostructures. Rough surfaces are clearly visible. The MoS_2_ membrane consists of many randomly orientated nanosheets (NSs) that cover the entire substrate. Each sheet consisted of the stacked MoS_2_ atomic layers with the extent of sheet overlap heavily influenced by the deposition time. As shown in Figure 1a, the average diameter of each continuous sheet is around 3~4 µm. The binary MoS_2_-TiO_2_ sample was then coated with nano ZnO to form ternary materials.

Figure 1b illustrates the surface morphology of ZnO after the coating of the binary sample to form a ternary nanoheterostructure (NHS). The ZnO layer comprises numerous 1D wires randomly distributed over the MoS_2_ surface. Each wire’s average length and diameter are 40 µm and 0.5 µm, respectively. A high-resolution TEM was used to directly observe the fine fringes at the edges of the 2D MoS_2_ cluster. Each fringe corresponds to a single atomic layer with a thickness of approximately 0.65 nm, allowing the thickness of the resulting 2D MoS_2_ sheet to be estimated.

As observed from the SEM image, the p-type TiO_2_ nanoparticles were discretely distributed on the surface of the Si substrate. After depositing MoS_2_ sheets and n-type ZnO, we were unable to observe their interfaces directly. The randomly oriented ZnO wires are tightly packed within the MoS_2_ nanostructure, resulting in a unique heterostructure that significantly enhances the gas sensing capabilities. Due to their irregular shapes, the ZnO wires provide a large exposure surface area, a desirable high-performance feature for prototypes.

### 3.2. Composition and EDX Measurements

The samples’ chemical composition was examined using energy-dispersive X-ray Spectroscopy (EDX); a representative result is shown in Figure 1c. The peak at 50 eV is associated with oxygen (O), while the peaks at binding energies of 100 eV, 860 eV, and 960 eV are attributed to zinc (Zn). The peak observed at 230 eV corresponds to molybdenum disulfide (MoS_2_); the peak at 455 eV is attributed to titanium (Ti), and the peak at 180 eV originates from the silicon (Si) substrate.

The EDX analysis reveals notable changes after the deposition of ZnO nanowires (NWs). Specifically, the Si peak from the substrate is significantly attenuated, and the Ti signal becomes undetectable. Interestingly, the sulfur-to-molybdenum atomic ratio (S/Mo) in the MoS_2_ sheets slightly exceeds two, indicating the presence of molybdenum vacancies in the 2D sheet samples. These molybdenum vacancies are known to facilitate the dominant hole of transport, introducing acceptor-like states and promoting p-type conductivity in the material.

Furthermore, detecting Zn and O confirms the successful growth of ZnO on the substrate. Notably, the atomic ratio of Zn to O is approximately three, suggesting oxygen-deficient ZnO. This deficiency is significant because oxygen vacancies in ZnO can act as electron donors, imparting n-type conductivity to the material. Such oxygen-deficient ZnO is well recognized for its potential in enhancing electron transport and contributing to a range of electronic and optoelectronic applications. The combination of p-type MoS_2_, influenced by molybdenum vacancies, and n-type oxygen-deficient ZnO suggests the formation of a heterojunction interface, which directly influences charge transport and the overall electrical behavior of the system.

### 3.3. Raman and FTIR Measurements

Raman spectrum measurements were performed using a T64000 Raman spectrometer (JY-Horiba, HORIBA France S.A.S., Palaiseau, France) featuring a triple monochromator in subtractive mode and a CCD detector. An Olympus microscope with an 80X objective in backscattering mode was employed for focusing. The sample was excited by a laser (Coherent Argon Innova 70C, Coherent, Inc., Santa Clara, CA, USA) at a wavelength of 514.53 nm with 5 mW of power. Figure 1d displays the Raman spectra over the 300–700 cm^−1^ spectral range of as-synthesized MoS_2_-TiO_2_ after ZnO deposition. The Raman peak at 519 cm^−1^ is attributed to TiO_2_, consistent with the characteristic A1g band of the anatase TiO_2_ phase. A significant reduction in the TiO_2_ Raman signal confirms the abundance of MoS_2_ sheets and ZnO on the top overlayer.

Both Raman active $E_{2g}^{1}$ at 382.1 cm^−1^ and A_1g_ at 405.5 cm^−1^ modes are observed, respectively, indicting the presence of the MoS_2_ phase. The narrow Raman active $E_{2g}^{1}$ peak indicates a low defect concentration in the obtained MoS_2_. This is in good agreement with the quantitative chemical composition data obtained from the EDX analysis. The wavenumber difference between the $E_{2g}^{1}$ and the A_1g_ bands is 23 cm^−1^, suggesting that the average MoS_2_ sheet thickness is only a few atomic layers thick [29,31]. A Raman peak at 450.2 cm^−1^ is also observed. According to the literature [32,33], this peak is commonly assigned to the MoS_2_ 2LA mode, indicating that the ZnO Raman spectral line E_2_ (high) mode overlapped with the MoS_2_ 2LA mode. Furthermore, the Raman peak at 343.3 cm^−1^ is also clearly visible which confirms ZnO deposition.

The shift in the E_2_ (high) mode of the ZnO crystal could be explained by the different magnitude of the stress between the ZnO and MoS_2_ layers. Decremps and Pellicer-Porres [34] indicated that the E_2_ mode of the wurtzite ZnO crystal would shift to a higher frequency under the biaxial compressive stress within the c-axis-oriented ZnO. Ribut et al. [35] reported that the E_2_ (high) mode’s frequency shifts up to 457cm^−1^ for ZnO on sapphire and 475 cm^−1^ on PPC substrates, whereas the deposition of nano ZnO onto bulk ZnO crystals resulted in its Raman E_2_ (high) mode shift to lower frequencies, around 437 cm^−1^ [36]. Interactions between the two layers and the presence of stress could be among the reasons affecting spectral shifts.

FTIR spectra were taken in the transmission mode of the MoS_2_-TiO_2_ sample after ZnO deposition, as shown in Figure 1e. A characteristic peak around 501.5 cm^−1^ from 2D MoS_2_ shifts approximately 12.7 cm^−1^ towards a lower wavenumber at 488.8 cm^−1^ after ZnO deposition. Secondly, the spectral line at this peak appears to have a slightly asymmetric profile. Thirdly, the FTIR signal intensity shows a slight reduction following ZnO deposition, which is attributed to the light absorption by the ZnO overlayer.

Given the porous nature of the structure, significant light absorption is unlikely, indicating the presence of defective concentrations or contamination. The EDX spectra revealed a high Zn/O ratio of up to three, suggesting the presence of oxygen vacancies. Additionally, weak carbon components were detected in both samples, likely due to contamination during synthesis and sample transfer between chambers. As expected, the ZnO FTIR peak at 1011.6 cm^−1^ is observed in the ternary sample; however, the TiO_2_ FTIR signal was too weak to be identified.

### 3.4. Fabrication of the Prototype and Its Basic Electrical Current–Voltage Properties

**Figure 2:**
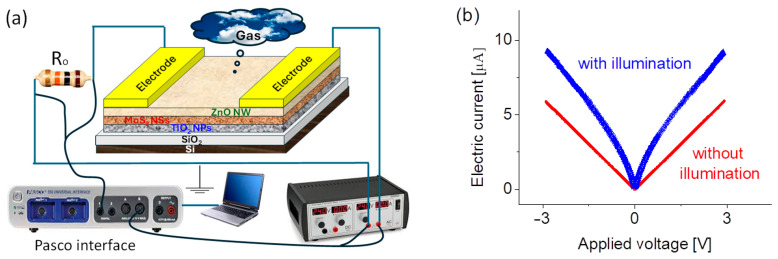
(**a**) Schematic of the prototype and its circuit and (**b**) Current–voltage (I–V) properties of the prototype with and without λ = 450 nm light illumination and with a power density of 5 mW/cm^2^.

Following the initial characterizations, a gold (Au) electrode array was deposited. The schematic of the prototype is detailed in Figure 2a. Briefly, a standard mask was placed on the top of the sample, and a DC plasma sputtering was used to deposit a pair of Au electrodes, each with a diameter of 4 mm and a thickness of approximately 100 nm. Then, the active layer was connected to a simple electric circuit to form a prototype. The sensor array design featured an active area of 1.2 cm^2^ between two electrodes, strategically optimized to enhance the interaction with target gas molecules. This configuration significantly improved the sensitivity and efficiency of the device. The integration of these well-defined gold electrode arrays plays a crucial role in connecting the nanostructured sensing material to macroscopic electronic circuits, enabling reliable signal transduction for advanced sensing applications.

The current–voltage (I–V) properties were evaluated using a PASCO 850 universal interface (PASCO Scientific, Roseville, CA, USA) and an HP 6212C power supply (Keysight Technologies, Inc., Santa Rosa, CA, USA). Characterizations were performed under an open-air atmospheric condition. Figure 2b shows the current–voltage properties with and without λ = 450 nm light illumination and with a power density of 5 mW/cm^2^. Linear current–voltage curves are clearly visible, indicating that the contact resistance or interface states may be influencing charge transport, leading to a lower barrier for carrier injection. Possible explanations include high defect density or Fermi-level pinning at the heterointerface, which can reduce or eliminate the expected rectification effect. Another possibility is the formation of intermediate states or tunneling-assisted conduction, which can result in a linear I–V response despite the presence of a heterojunction. If a true heterojunction exists, its built-in electric field should separate photogenerated electron–hole pairs, leading to a noticeable nonlinear change in the I–V curve with illumination. Once a voltage is applied across the electrodes at the two ends of the active layer, the induced electric field separates the pair, resulting in electrons drifting toward the electrodes and contributing to increased conductivity. Additionally, temperature-dependent measurements revealed that the resistance of the active layer decreased from 225 kΩ to around 157 kΩ as the operating temperatures increased from 25 °C to 100 °C and 150 °C. This trend suggests enhanced charge carrier mobility at elevated temperatures, further influencing the sensor’s electrical properties.

### 3.5. Sensor Performance

The gas sensing prototypes were fabricated based on ZnO-MoS_2_-TiO_2_ ternary NHS. The performance of the prototypes was characterized with a 5 V bias at room temperature. The target gas concentrations were regulated using two MKS GE50A mass flow controllers, with monitoring carried out through two MKS 946 vacuum system controllers. Once the target gas outlet valve was opened, a set concentration of the target gas was introduced into a 1 L chamber. Given that the nitrogen gas flow rate into the chamber was maintained at 500 sccm and the flow rate of 100 ppm NO_2_ gas from the tank to the chamber was 20 sccm, it was anticipated that achieving a stable concentration of 4 ppm within the chamber would require at least 2 min.

**Figure 3:**
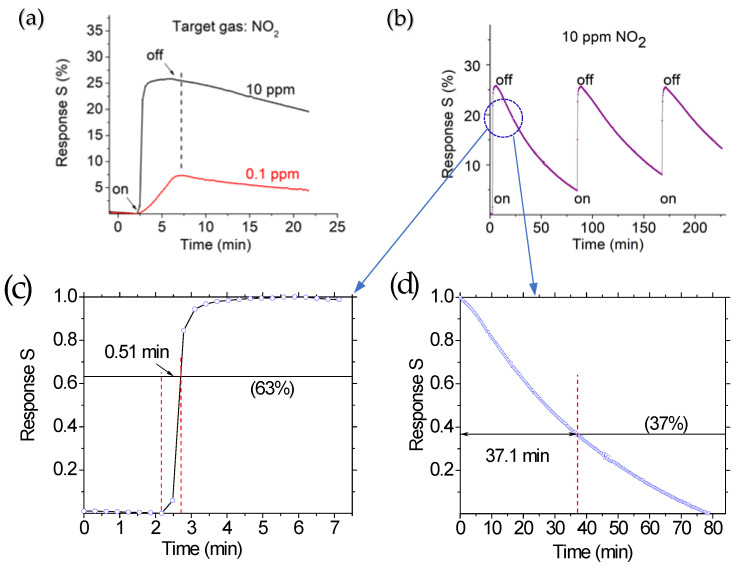
(**a**) Room-temperature responses of the ZnO-MoS_2_-TiO_2_-based prototype exposed to 10 ppm and 0.1 ppm NO_2_ gas. (**b**) Repeatability of the prototype exposed to 10 ppm NO_2_ target gas. (**c**) Response and (**d**) recovery time.

Figure 3a shows the response of the prototype when exposed to NO_2_ gas at concentrations of 10 ppm and 0.1 ppm at room temperature. The response strength is defined as the ratio of the current change (ΔI) caused by the target gas to the initial current of the prototype in air. Mathematically, this is expressed as S = ∆I/I_o_ = (I_s_ − I_o_)/I_o_, where I_s_ represents the current in the presence of the target gas, and I_o_ is the initial current of the prototype in the air. The adsorption of NO_2_ gas onto the surfaces of the active layers through chemical bonding and electron transfer results in a change in resistance.

It should be noted that the prototype’s characterizations were carried out at room temperature. The obtained response time was relatively long before reaching its steady state. Several studies have explored and discussed the factors and mechanisms responsible for the response and recovery times. Still, most of the reported gas sensors operating at room temperature exhibited very long response times of up to 60 min [37,38,39,40]. For instance, Moumen et al. documented recovery times of up to 4000 s for room-temperature NO_2_ sensors, while Kok et al. discussed possible mechanisms and influencing factors [41]. Wang et al. proposed the use of heterojunctions between transition metal dichalcogenides and ZnO nanoparticles to improve NO_2_ sensor recovery time [42]. Under room-temperature conditions, the recovery time is influenced not only by the properties of the active layer but also by the experimental setup such as the gas flow rate and the test chamber size, which affect gas diffusion after the target gas inlet valve is turned off. A slight increase in operating temperature is expected to improve sensing features for both response strength and response time, but this would also inevitably result in a complicated structure requiring an additional heater and power supply.

Another factor that led to a long recovery time was the gas flow rate and test chamber size involved in gas diffusion after the inlet valve for the target gas is turned off. To illustrate this effect, a simple mathematical model can be used. In the present setup, the N_2_ gas flow rate is 180 sccm, and the 100 ppm NO_2_ gas flow from the tank to the chamber is 20 sccm. The test chamber dimensions are 10 × 10 × 10 cm³. If experiments are conducted at a concentration of 10 ppm NO_2_, it will take approximately 11.5 min for the concentration inside the chamber to decrease from 10 ppm to 1 ppm after the 100 ppm NO_2_ inlet valve is closed.

Using a smaller test chamber can largely mitigate this delay. Using a smaller test chamber can significantly reduce this delay. For example, if the chamber volume is reduced to approximately 100 cm³ with a proportionally increased flow rate, the characteristic dilution time decreases to about 30 s. Under such optimized conditions, response times as short as 20 s and recovery times of 60–70 s have been achieved [43]. Therefore, the actual response and recovery times may be shorter than they appear in conventional setups. Ideally, to accurately measure the response time, the chamber should first be stabilized at a specific gas concentration before inserting the sensor. For an accurate recovery time measurement, the sensor should remain inside the chamber at a known concentration before being removed into ambient air, where its signal decay can be immediately recorded.

It was noticed that the lower the concentration of the target gas, the longer it takes to reach a stable state, as shown in Figure 3a. The response time parameters are influenced by the adsorption and desorption of target gas molecules on the active layers, which depend on both the gas concentration and the operating temperature. After the outlet valve was opened for 30 s, the response signal strength reached 0.264 for the target gas concentrations of 10 ppm. Since the time to reach the balance of the desired concentration of the target gas inside the chamber was around 2 min, the expected response strengths would be much larger than 0.264 after the gas concentration inside the chamber reached 10 ppm. Clearly, the response of the ternary NTS-based prototype is very fast, less than 1 min. A similar phenomenon was also observed at a concentration of 0.1 ppm. After the outlet valves were opened for 4 min, the response signal strength was 0.076.

Figure 3b shows the sensor’s repeatability when exposed to NO_2_ gas at a concentration of 10 ppm. The response time (0.51 min, Figure 3c) and response strength (0.26) remained almost unchanged over three cycling tests. However, the recovery time was notably long, reaching up to 37.1 min (Figure 3d). The recovery time is defined as the duration it takes for the sensor signal to drop to 1/e ~ 37% of its original peak value, while the response time is the time taken for the output to reach (1 − 1/e) ~ 63% of its maximum value. The actual response and recovery times may be shorter, as delays occur in reaching the desired concentrations after turning the target gas source on or off. This indicated that the desorption of NO_2_ molecules from the active layer surface was slow at room temperature.

**Figure 4:**
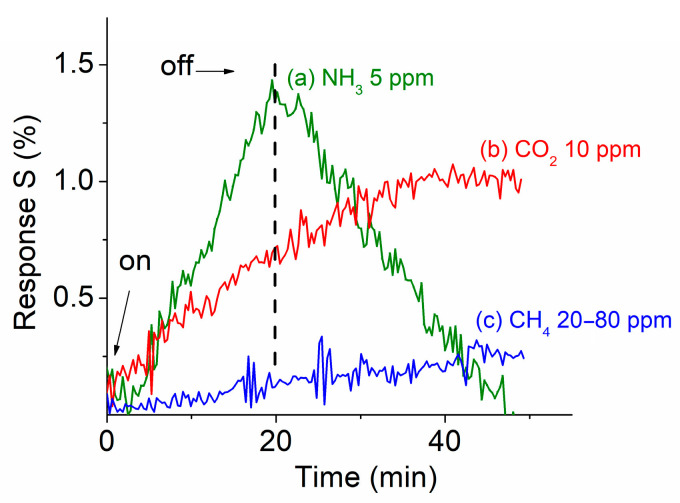
Selectivity of the ternary NHS prototype at room temperature when exposed to (a) NH_3_, (b) CO_2_, and (c) CH_4_ gases at different gas concentrations.

To understand the selective properties of the fabricated prototype, additional experiments were also carried out with the ZnO-MoS_2_-TiO_2_-based prototype exposed to three other target gases. Figure 4 shows the selective response properties of the ZnO-MoS_2_-TiO_2_ prototype when exposed to (a) ammonia (NH_3_), (b) carbon dioxide (CO_2_), and (c) methane (CH_4_) at different concentrations. The inlet valve of the target gas was opened at t = 0 and then closed after 20 min for all three gases. The prototype showed almost no detectable response signal for CH_4_ gas even at concentrations up to 80 ppm and only a very weak response for CO_2_ gas at a concentration of 10 ppm. In contrast, a clear response signal was observed from the prototype when exposed to NH_3_ even though its signal was modest. At a concentration of 5 ppm NH_3_, the obtained response strength reached up to 0.015. A stronger response signal was observed with increased exposure time, as shown in Figure 4.

As is well known, a long minority carrier lifetime enhances the response strength and slows down the response speed, creating a fundamental trade-off between signal strength and the response speed. Typically, minority carrier time in doped materials relies on the capture rate for holes and electrons at the recombination centers. The response time can be affected by many factors, including doping concentrations, the configuration of hybrid nanostructures, interface junctions, and the characteristics of the depletion region. Therefore, controlling the hybrid structure and composition is crucial for achieving a fast response time without sacrificing too much response strength. Optimizing hybrid nanostructures and minimizing this trade-off to achieve a desired balance of response speed and response strength for high-performance sensing devices is one of the primary objectives of this work. Other potential strategies to balance NO_2_ sensitivity with faster recovery include optimizing defect density and heterostructure interfaces to improve charge transport and minimize charge trapping. Controlled annealing can reduce oxygen vacancies in ZnO and TiO_2_, while fine-tuning the ZnO-MoS_2_-TiO_2_ composition and defect states can help mitigate deep-level charge trapping, ultimately enhancing desorption kinetics.

**Figure 5:**
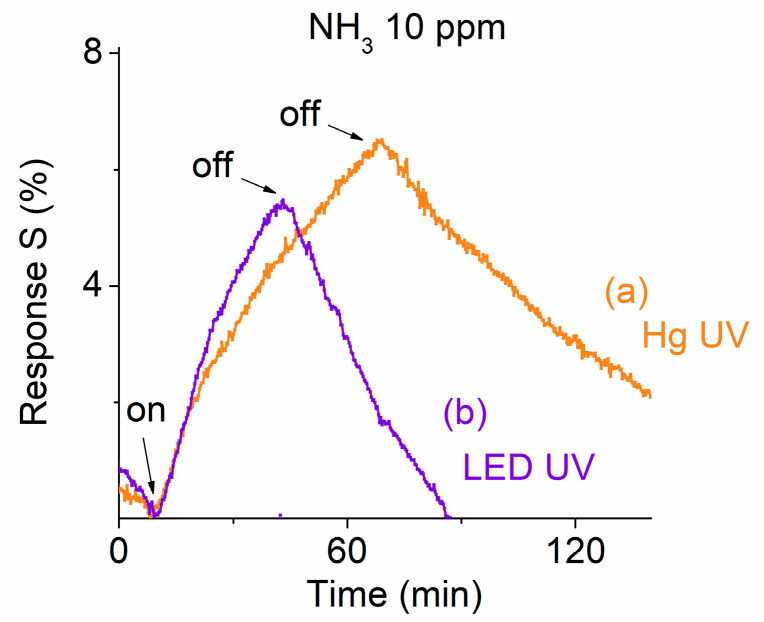
Responses of the ternary NHS prototype at room temperature when exposed to 10 ppm NH_3_ gas under (a) Hg UV and (b) LED UV illuminations.

Light-enhanced gas sensors utilize illumination to generate additional charge carriers, thereby improving sensitivity and response times. Therefore, we conducted light-enhanced gas sensing tests on the ternary NHS prototype. Initially, a commercial mercury (Hg) UV light (1 mW/cm^2^, λ = 300–365 nm) was used. Figure 5(a) presents the response of the ternary NTH-based prototype exposed to 10 ppm NH_4_ gas. However, commercial mercury UV lights are generally too large for compact sensing devices and require high-voltage power supplies. To facilitate miniaturization, a small LED UV light (6 mW/cm^2^, λ = 365 nm) powered by a 1.5 V battery was also used to assess its effect on the gas sensor response.

Figure 5(b) presents the responses of the ternary NTH-based prototype exposed to 10 ppm NH_4_ gas under LED UV illumination. The results indicate that both Hg UV light and LED UV light can enhance the prototype’s sensitivity. The obtained response strength under LED UV illumination appeared slightly better than that with Hg UV light. After turning on the inlet valve of the target gas to the chamber for 30 min, the obtained response is around 0.052 under LED UV and around 0.047 under Hg UV illumination. It suggests that LED UV illumination may provide higher efficiency in enhancing the response strength. Given that the wavelengths of the LED and Hg UV lights are close, around λ = 365 nm, but have a tenfold difference in light intensity, it appears that a stronger UV intensity correlates with a higher response strength. To fully understand the light illumination effect, further experiments were also carried out where different wavelengths of LED lights were applied.

**Figure 6:**
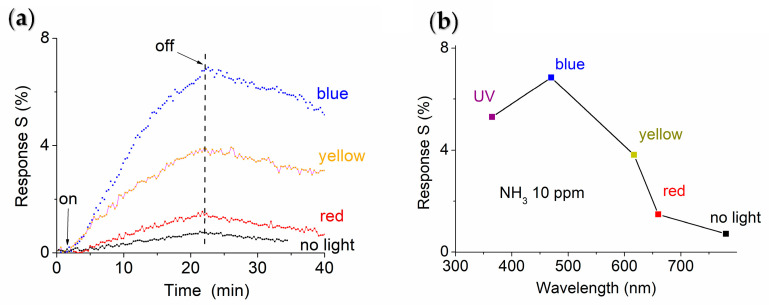
(**a**) Room-temperature response of the ternary NTH prototype to 10 ppm NH_3_ gas under 6 mW/cm^2^ LED illumination at various wavelengths. (**b**) The response strength (S) as a function of illumination wavelengths.

Figure 6 shows the responses of the ternary NTH prototype exposed to 10 ppm NH_3_ gas with and without 6 mW/cm^2^ LED light illumination in blue (470 nm), yellow (570 nm), and red (625 nm). It is evident that visible light illumination enhances the response signal. Without LED light illumination, the obtained response strength is only 0.0075, as shown in Figure 6a. In contrast, under red-, yellow-, and blue-light illuminations, the response strengths increase to 0.014, 0.038 and 0.068, respectively. Figure 6b shows the response strength as a function of illumination wavelength. UV and blue-light LED illumination yielded a better performance than those illuminations at longer wavelengths.

It should be mentioned that the experiments have been replicated multiple times during the last 12 months, and the obtained data including the response time and the response strength exhibit remarkable stabilization. This stability ensures that the sensor maintains a consistent and reliable signal over time. The effects from different humidity levels from 20% to 80% have been investigated. If the target gas concentration was above 1 ppm, no significant humidity effect was observed. This is in line with the outcomes of other studies [44]. A detailed investigation of the humidity effect can be found in our previous work [45].

The experiments were also carried out under an inert nitrogen atmosphere and under open air. Before introducing the pollution gas into the test chamber, the response (either resistance or current) of the prototype remained nearly identical across these two conditions. However, once NO_2_ pollution gas was introduced into the chamber, the resistance of the active layer changed because of the variation in the depletion region. The experimental data showed that the obtained resistance under air was slightly larger (around 5%) than that under an inert nitrogen atmosphere. The fabricated prototype demonstrated an exceptional performance including outstanding selectivity (response ratio > 17) and a short response time less than 0.5 min as well as a 58% sensitivity at 4 ppm and 6% at 0.1 ppm of NO_2_ as compared with previous work [46].

Table 1 compares the performance of room-temperature NO_2_ gas sensors based on TiO_2_, MoS_2_, and ZnO monocomponent, binary and ternary nanoheterostructures reported in the literature, in terms of the limit of detection (LOD), response time, and recovery time. Evidently, the monocomponent nanomaterial-based sensors exhibit limited baseline sensitivity and responsivity, as determined by the inherent properties of the single material. The binary nanoheterostructure-based sensors offer improved sensitivity, responsivity, and response times compared to monocomponent materials. Our proposed ternary sensor shows the highest sensitivity and responsivity, as well as the fastest response time among the three categories. In addition, our ternary sensor offers excellent selectivity.

### 3.6. Sensing Mechanisms

EDX data indicated that the atom ratio of O/Ti in TiO_2_ layers is significantly greater than two (O/Ti~40); the S/Mo ratio in MoS_2_ layers is very close to two (S/Mo~2), and the O/Zn ratio in ZnO NWs is much less than unity (O/Zn << 1). Therefore, low hole (h^+^) carrier concentrations in MoS_2_, high hole (h^+^) carrier concentrations in TiO2, and high electron (e^−^) carrier concentrations in ZnO are expected. A high electron carrier concentration is usually related to many charge carriers that exhibit enhanced sensitivity to external stimuli, which is beneficial for sensor applications. The conductivity of ZnO nanowires is n-type, whereas that of TiO_2_ is p-type. This p-n junction configuration significantly increases the sensitivity of gas sensors. Direct exposure to target gases is provided by the significant number of quantum dots and one-dimensional quantum wells that form at the interfaces of MoS_2_ nanosheets, TiO_2_ NSs, ZnO NWs, and MoS_2_ NSs. Furthermore, a structure with a porous-like morphology is produced by the partial overlap of ZnO NWs, MoS_2_ NSs, and TiO_2_ NSs.

**Figure 7:**
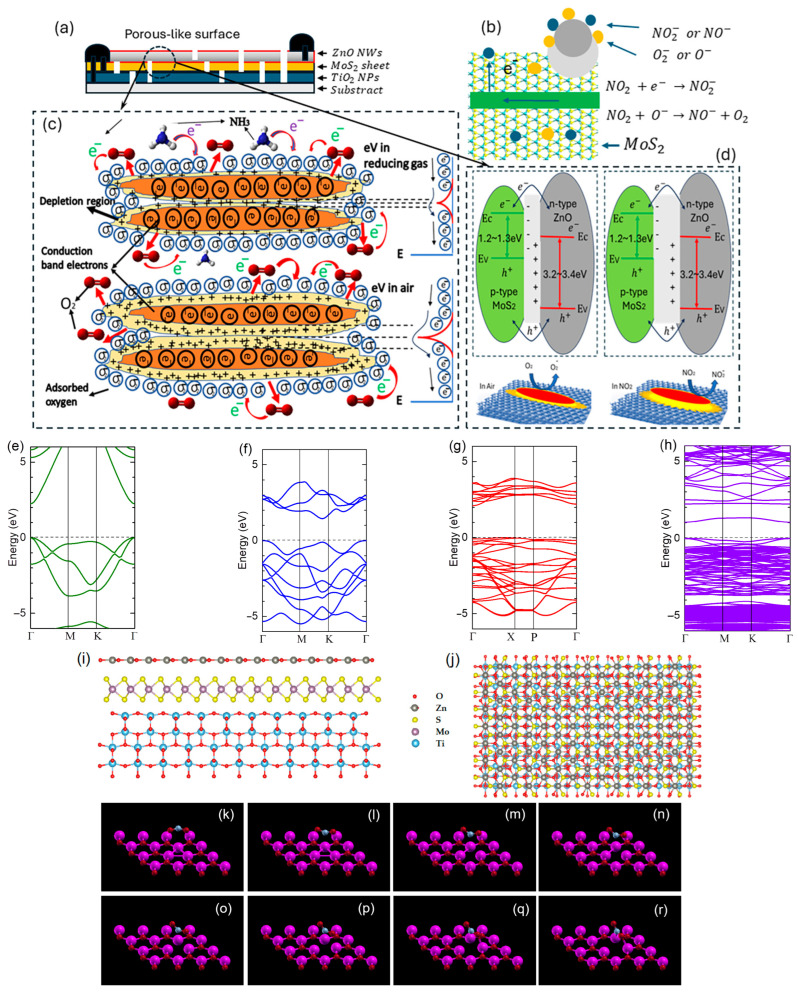
(**a**) Schematic of the ternary ZnO-MoS_2_-TiO_2_ NHS prototype. (**b**) The interaction of NO_2_ gas molecules with the ternary NHS prototype. NO_2_ molecules adsorb on the NHS’s surface, leading to electron transfer between NO_2_ molecules and the NHS components. This process creates ${NO}_{2}^{-}$ or ${NO}^{-}$ adsorbates on the surface. (**c**) The energy band alignment at the MoS_2_ (green lines) and ZnO (red lines) interface for NH_3_ sensing. (**d**) The energy band alignment at the MoS_2_ (green lines) and TiO_2_ (blue lines) interface for NO_2_ sensing. Calculated band structures of (**e**) ZnO, (**f**) MoS_2_, (**g**) TiO_2_ monolayer, and (**h**) ZnO with NO_2_ adsorbed. (**i**) Side and (**j**) top view of ternary heterostructure. (**k**–**r**) DFT simulations of the interaction sequence of NO_2_ adsorption over the ZnO surface.

Enhancing NO_2_ sensitivity in this work requires the ternary nanoheterostructure, which integrates semiconductor materials with complementary characteristics. Multiple charge transfer channels and improved gas molecule adsorption sites are used to accomplish this enhancement. The hybrid structure facilitates effective interactions between NO_2_ molecules and the active sensing regions by utilizing its constituent parts’ distinct electrical and surface properties. As depicted in Figure 7a, one electrode is positioned on the MoS_2_ nanosheets (partially overlapping the TiO_2_ nanoparticles), while the other electrode is placed on the ZnO nanowires (partially on MoS_2_). This electrode configuration ensures a well-distributed interaction between the different materials in the heterostructure, enhancing charge mobility and response uniformity across the sensing layer. To create porous, three-dimensional architectures made of interconnected 0D/1D/2D-active materials, it is essential to efficiently manage the hybrid structure’s morphology, composition, and density of nanowires and nanosheets. Because electron-depleted zones are extremely susceptible to gas adsorption, these structures are necessary for their formation. The responsiveness and selectivity of the sensor are strongly impacted by the capacity to precisely control these electron-depleted zones, mainly when operating at room temperature. The 3D porous-like network also ensures enhanced gas diffusion and interaction, which further amplifies the sensor’s performance, making it highly suitable for practical NO_2_ detection applications.

Figure 7a presents the side view of the sensor structure. Figure 7b illustrates the NO_2_ gas sensing mechanism and charge transfer dynamics, where ZnO provides a high electron concentration, while MoS_2_ facilitates hole conduction. To create ${NO}_{2}^{-}$ or NO^−^ adsorbates, NO_2_ gas molecules draw electrons from the NHS when they adsorb onto the surface of nanostructured ZnO NWs. The NHS’s conductivity is changed by this electron removal, producing a measurable sensing signal. Likewise, oxygen (O_2_) creates adsorbates by adsorbing them on the sensor’s surface. As a result, oxygen adsorbates oxidize the molecules of the external gas, returning the electrons to the sensor and altering its conductivity. In contrast, NH_3_ is a reducing gas, meaning its adsorption reduces the depletion region by donating electrons to the sensor (Figure 7c). This electron injection lowers resistance and alters the sensor response. Conversely, NO_2_ is an oxidizing gas, increasing the depletion region when adsorbed onto the active layers, leading to higher resistance (Figure 7d). Charge transmission is facilitated, and the band alignment at the interfaces of these materials influences the NHS’s total conductivity. The response of the sensor is directly impacted by variations in operating temperatures and gas concentrations.

Furthermore, the NHS’s surface morphology, including its vacancies, flaws, and active sites, is essential to charge transfer and NO_2_ adsorption. Numerous quantum dots and 1D quantum wells were created at the interfaces due to 3D porous constructions. The conductivity of MoS_2_ is increased when external gas molecules adsorb on the surfaces of nanostructured zinc oxide. This occurs because the electrons from the sensor will move in the direction of the gas adsorbates, attracting additional electrons that are moving from MoS_2_ to ZnO. Such a structure achieved high mobility of electron transport and electronically increased gas sensitivity. The surface morphology of the THS, including vacancies, flaws, and active sites, plays a crucial role in charge transfer and NO_2_ adsorption. The 3D porous structure facilitates the formation of numerous quantum dots and 1D quantum wells at the interfaces, enhancing charge transport. When external gas molecules adsorb onto the nanostructured ZnO surface, the conductivity of MoS_2_ increases due to electron transfer. This occurs as electrons migrate toward the gas adsorbates, attracting additional electrons from MoS_2_ to ZnO. This design enables high electron mobility and significantly enhances gas sensitivity.

Density functional theory (DFT) calculations have been utilized to study the adsorption behavior of the ternary ZnO-MoS_2_-TiO_2_ heterostructure-based NO_2_ sensor. First-principles calculations were carried out with the Quantum Espresso (QE) 7.3 package. Generalized gradient approximation (GGA) with the Perdew–Burke–Ernzerhof (PBE) functional was utilized as the exchange correlation functional to determine the geometric structures and electronic properties. The pseudopotentials were described using the projected augmented wave (PAW) method, and the valence configurations are as follows: Zn (3d^10^4s^2^), O (2s^2^2p^4^), Mo (4s^2^4p^6^4d^4^5s^2^), S (3s^2^3p^4^), Ti (3p^6^3d^2^4s^2^), and N (2s^2^2p^3^). To help accurately describe the weak van der Waals (vdW) interactions between layers, the DFT-D3 correction by Grimme was used.

To obtain accurate geometric and electronic properties, an energy cutoff of 680 eV, an energy convergence tolerance of 1.0 × 10^−6^ eV, convergence thresholds for atomic force of 10^−2^ eV/Å, and a Monkhorst–Pack k-point grid of 7 × 7 × 1 were found to be sufficient. A vacuum space thickness of 15 Å was used between neighboring cells in the c-axis to simulate a monolayer. All structures were fully relaxed until the Hellmann–Feynman force on each atom was <0.1 eV/Å. The ZnO with vacancy was relaxed both independently before the introduction of NO_2_ and then again with NO_2_ adsorbed. The detailed simulation method was reported in Ref [52].

The investigation focused on adsorption mechanisms, charge transfer, and electronic structure modifications upon NO_2_ interactions. Results indicate strong chemisorption at bridge sites, facilitated by charge redistribution between Zn donor sites and NO_2_ acceptor states. Band structure and density of states (DOS) analyses reveal significant changes in electronic properties, highlighting the heterostructure’s potential as a high-sensitivity NO_2_ sensor. The calculated electronic structures and adsorption characteristics provide insights into the sensing potential of the ZnO-MoS_2_-TiO_2_ heterostructure for NO_2_ and other gas detections. Figure 7e–h depict the band structures of ZnO-, MoS_2_-, TiO_2_-, and NO_2_-adsorbed ZnO, respectively, highlighting how adsorption modifies the electronic properties. In these figures, the valence band maximum is set to zero. The band structure changes upon NO_2_ adsorption indicate a shift in the Fermi level and a reduction in the bandgap, a key factor for sensing applications. Figure 7i,j illustrate the side and top views of the schematic diagram of the ternary ZnO-MoS_2_-TiO_2_ heterostructure. For simplicity, the 2D nanomaterials were considered as monolayers with interlayer spacings of 0.285 nm between TiO_2_ and MoS_2_ and 0.292 nm between MoS_2_ and ZnO, respectively. Figure 7k–r display the interaction sequence of NO_2_ molecules with the ZnO surface, showing preferred adsorption sites and charge redistribution. These structural deformations and electronic modifications confirm strong chemisorption, facilitating charge transfer and making the heterostructure highly responsive to NO_2_. The findings suggest that the ZnO-MoS_2_-TiO_2_ heterostructure is a promising candidate for NO_2_ sensing due to its improved adsorption properties and electronic response.

UV light can further improve sensor responsiveness by encouraging the adsorption of NO_2_ molecules on the NHS surface. It facilitates the breakdown of NO_2_ bonds with increased adsorption, generates electron–hole pairs during the sensing process, and activates surface sites and defects to make them more reactive towards NO_2_ molecules. Furthermore, UV light can assist in the desorption of NO_2_ molecules from the surface, reducing sensor recovery time and enabling a quick return to baseline signal levels after exposure to NO_2_.

## 4. Conclusions

The experimental data obtained provided strong evidence of the high sensitivity of the ZnO-MoS_2_-TiO_2_ prototype for NO_2_ detection. It is significantly better in detection limit and response time than previously reported when operating at room temperature. During three cyclic tests, the response time (0.5 min) and the response intensity (0.26) remained almost unchanged. However, the recovery time is relatively long, up to 35 min, indicating a slow desorption of NO_2_ molecules from the surface of the active layer at room temperature. Studies of the prototype’s selective properties showed that there was almost no detectable response signal for CH_4_ gas at concentrations up to 80 ppm and only a weak response for CO_2_ gas at concentrations of 10 ppm. A clear response signal was found when the prototype was exposed to NH_3_, although the signal was not very strong at a concentration of 5 ppm. A fundamental trade-off appears between the response signal’s intensity and a detector’s response speed. Effective control of the hybrid structure and compositions is critical to minimizing this trade-off and achieving the desired balance for high-performance sensing devices. Experimental data from studies of the effects of ultraviolet, blue, yellow, and red LED illuminations on the prototype response indicated that illumination with light greatly enhances the response intensity. The prototype illuminated with blue LED light appeared to perform better than the prototype illuminated with light at shorter or longer wavelengths.

## Figures and Tables

**Table 1:** Comprehensive performance comparison of room-temperature NO_2_ gas sensors based on TiO_2_, MoS_2_ and ZnO monocomponent, binary and ternary nanoheterostructures.

Material	LOD [ppm]	Response Time	Recovery Time	Ref.
MoS_2_	5	~80 s	2 s	[47]
TiO_2_	10	35 s	18 s	[48]
ZnO	5	20 min	15 min	[49]
ZnO-MoS_2_	0.5	>40 s	-	[50]
TiO_2_-ZnO	5	26/36 s	224/271 s	[51]
ZnO-MoS_2_-TiO_2_	0.1	31 s	37 min	This work

## Data Availability

Data underlying the results presented in this paper are available from the authors upon request.
